# Is adjuvant chemotherapy beneficial for patients with FIGO stage IC adult granulosa cell tumor of the ovary?

**DOI:** 10.1186/s13048-018-0396-x

**Published:** 2018-03-27

**Authors:** Dan Wang, Yang Xiang, Ming Wu, Keng Shen, Jiaxin Yang, Huifang Huang, Tong Ren

**Affiliations:** Department of Obstetrics and Gynecology, Peking Union Medical College Hospital, Chinese Academy of Medical Science and Peking Union Medical College, No. 1 Shuaifuyuan Road, Dongcheng District, Beijing, 100730 People’s Republic of China

**Keywords:** Adult granulosa cell tumor, Adjuvant chemotherapy, Stage IC

## Abstract

**Background:**

To evaluate the association between adjuvant chemotherapy and clinical outcomes in patients with stage IC adult granulosa cell tumor (AGCT).

**Methods:**

We performed a retrospective study of patients with stage IC AGCT diagnosed at our hospital from January 1985 to September 2015. We analyzed descriptive statistics, and performed univariate and multivariate and Kaplan–Meier survival analyses.

**Results:**

Sixty stage IC AGCT patients were identified, including 28 in the no adjuvant chemotherapy group (NACG) and 32 in the adjuvant chemotherapy group (ACG). The median follow-up time was 88 months (range: 9–334 months). Sixteen patients developed recurrences, including nine in the NACG and seven in the ACG groups. Univariate analysis identified incomplete surgical staging and initial treatment place as associated with disease-free survival (DFS) (*P* = 0.003 and 0.038, respectively). Incomplete surgical staging remained a risk factor for recurrence in multivariate analysis (hazard ratio (HR) = 3.883, 95% confidence interval (CI): 1.123–13.430, *P* = 0.032). The 5-year DFS rates in the NACG and ACG groups were 76.3% and 87.5% respectively (*P* = 0.197). Adjuvant chemotherapy was thus not associated with improved DFS. Furthermore, the number of chemotherapy cycles was not associated with recurrence rate (≤3 cycles vs. > 3 cycles, HR = 0.613, 95% CI: 0.112–3.351, *P* = 0.572).

**Conclusion:**

Administration of adjuvant chemotherapy does not improve DFS in patients with stage IC AGCT. Further studies with larger samples involving multi-institutional collaboration are needed to validate new treatment regimens for this disease.

## Background

Granulosa cell tumors (GCTs) are uncommon, accounting for only about 5% of all ovarian malignancies, but comprising 70% of ovarian sex cord-stromal tumors [1]. Most GCTs are adult GCTs (AGCTs), based on their clinical presentation and histological findings. AGCT comprises a clinically and molecularly unique subtype of ovarian malignancy with different behavior from other histological subtypes. The majority of AGCTs are diagnosed at an early stage and have a good prognosis, with 5- and 10-year overall survival rates of 98% and 84%, respectively [2]. However, AGCTs can occasionally be indolent, with a tendency to late relapse, associated with significant morbidity and difficult therapeutic choices.

Surgery is the cornerstone of treatment for AGCT, and patients with stage I AGCT have a favorable prognosis following surgical treatment alone, though the National Comprehensive Cancer Network guidelines (NCCN) recommend adjuvant chemotherapy for patients with advanced stage disease, or stage I disease with high risk factors. However, the definition of what constitutes a high risk factor remains unclear, and current evidence regarding the use of adjuvant chemotherapy in women with early stage AGCT is conflicting. Some studies have suggested that women might benefit from adjuvant chemotherapy [3, 4], while others failed to show any effect of postoperative chemotherapy on survival or relapse rates [5, 6]. This lack of clear evidence regarding the benefit of adjuvant chemotherapy in early stage AGCT makes treatment decisions difficult.

According to the revised FIGO stage (2014), ovarian epithelial cancer stage IC can be subdivided into intraoperative rupture (IC1), capsule ruptured before surgery or tumor on ovarian surface (IC2), and malignant cells in ascites or peritoneal washings (IC3). This new FIGO staging provides a more precise definition of the risk in stage IC [7]. Indeed, patients with stage IC have a higher relapse rate and shorter median time to relapse compared with stage IA patients [8, 9]. Some authors suggest the use of adjuvant therapy in AGCT stage IC patients with preoperative rupture or malignant ascites [10], but there remains limited information regarding the role of adjuvant chemotherapy in stage IC [8, 11]. The aim of this study was thus to evaluate the association between adjuvant chemotherapy and disease-free survival (DFS) in patients with stage IC AGCT.

## Methods

This study was approved by the ethics committee of our hospital. All patients diagnosed with AGCT at our hospital from January 1985 to September 2015 were reviewed. Sixty patients were diagnosed with FIGO stage IC AGCT.

Information was collected from all patients regarding age, menopausal status, tumor diameter, preoperative serum CA125, FIGO stage, type of surgery, adjuvant therapy, relapse characteristics, and relapse treatment and follow-up information. Follow-up information was obtained from outpatient files or by telephone interview with patients or their relatives. Tumor stage was based on the new staging system reports (FIGO staging system, FIGO Committee on Gynecologic Oncology, 2014) [7].

All patients underwent surgery. Fertility-sparing surgery was defined as preservation of the uterus and at least one ovary. Total abdominal hysterectomy and bilateral salpingo-oophorectomy was classified as radical surgery. Staging was considered complete when it included peritoneal washing, omentectomy (or omental biopsy), multiple peritoneal biopsies, and biopsy of any suspicious area. Pelvic and/or para-aortic lymphadenectomy were optional procedures, according to the surgeon’s experience and the intraoperative findings.

The exact indications for adjuvant chemotherapy in the present study were unclear because of the retrospective nature of the study, and the decision to administer adjuvant chemotherapy was made by the attending physicians after discussion with the patients.

### Statistical analysis

Statistical analysis was performed using SPSS version 15 (SPSS, Inc., Chicago, IL, USA). Patient demographics and baseline characteristics were summarized using descriptive statistics. Patients were defined into no adjuvant chemotherapy group (NACG) and adjuvant chemotherapy group (ACG). Median values were compared using Mann–Whitney U-tests and frequency distributions were compared using χ^2^ and Fisher’s exact tests. The main objective of the study was to evaluate the association between adjuvant chemotherapy and disease-free survival (DFS), defined as the time from initial surgery to the first recurrence or date of censoring. DFS survival curves were obtained using the Kaplan–Meier method and compared using log-rank tests. A *P* value < 0.05 was considered statistically significant. Variables with *P* < 0.05 on univariate analysis were selected for multivariate analysis.

## Results

Sixty patients with Stage IC AGCT were identified during the study period, including 28 in the NACG group and 32 in the ACG group. The median age at diagnosis was 41 years (range: 23–75 years). Their baseline characteristics are summarized in Table 1. The FIGO distributions were as follows: surgical spill in 34 patients (IC1), capsule ruptured before surgery or tumor on ovarian surface in 23 patients (IC2), and malignant cells in ascites or peritoneal washings in three patients (IC3).

**Table 1:** Clinical characteristics of AGCT in stage IC

	All patients	NACG	ACG	p
	*N* = 60	*N* = 28 (%)	*N* = 32 (%)	
Age (median)	41.0	41.5	40.5	0.410
Tumor size (cm)	8	7	8.4	0.523
Serum Ca125(U / ml)	15.5	14.4	16.4	0.751
Menopause				0.744
Yes	14 (23.3)	6 (21.4)	8 (25.0)	
No	46 (76.7)	22 (78.6)	24 (75.0)	
FIGO stage (%)				0.944
IC1	34 (56.7)	16 (57.1)	18 (56.2)	
IC2-IC3	26 (43.3)	12 (42.9)	14 (43.8)	
Surgical procedure				0.605
Fertility surgery	30 (50.0)	15 (53.6)	15 (46.9)	
Radical surgery	30 (50.0)	13 (46.4)	17 (53.1)	
Staging operation				0.102
Yes	26 (43.3)	9 (32.1)	17 (53.1)	
No	34 (56.7)	19 (67.9)	15 (46.9)	
Lymphadenectomy				0.245
Yes	24 (40.0)	9 (32.1)	15 (46.9)	
No	36 (60.0)	19 (67.9)	17 (53.1)	
First operation at clinical				0.696
Our clinical	37 (61.7)	18 (64.3)	19 (59.4)	
Outer	23 (38.3)	10 (35.7)	13 (40.6)	

AGCT: adult granulosa cell tumor; ACG: adjuvant chemotherapy group; NACG: no adjuvant chemotherapy group

All patients underwent upfront surgery, including 26 (43.3%) who underwent complete surgical staging and 34 (56.7%) who did not. Twenty-four patients (40%) had pelvic and/or para-aortic lymphadenectomy during surgery and the removed lymph nodes were all negative for metastatic AGCT. Thirty-seven (61.7%) patients received surgical treatment in our center and 23 (38.3%) were operated on elsewhere and then referred for subsequent evaluation postoperatively.

Thirty-two (53.3%) patients received adjuvant chemotherapy, with a mean of 3.2 (range: 1–6) chemotherapy cycles. The chemotherapy regimens included bleomycin, etoposide, and cisplatin (BEP) in 11 patients; cisplatin, vincristine, bleomycin in seven; cisplatin and cyclophosphamide in four; paclitaxel and carboplatin (TC) in five; and other regimens in five patients. Among the 32 patients who received chemotherapy, eight (25%) received more than three cycles and 24 (75%) received three or fewer cycles.

### Survival analysis

The median follow-up time was 88 months (range: 9–334 months). During the study period, sixteen patients (26.7%) experienced at least one recurrence, including nine in the NACG group and seven in the ACG group. Among all patients with recurrences, the median time to recurrence was 66 months (range: 7–165 months).

The anatomic locations of the first recurrences included the pelvis alone in eight patients, the abdomen alone in two, and the pelvis plus abdomen in six. Thirteen patients underwent debulking surgery plus chemotherapy and three patients received surgical reduction alone. Eleven patients (69%) developed a second recurrence (4 pelvic plus abdominal relapse; 3 pelvic; 3 abdominal; 1 hepatic involvement) after a median time of 48 months from diagnosis of the first recurrence (range: 24–105 months). Five patients were treated with surgery, five with surgery plus chemotherapy, and one with palliative care.

**Fig. 1:**
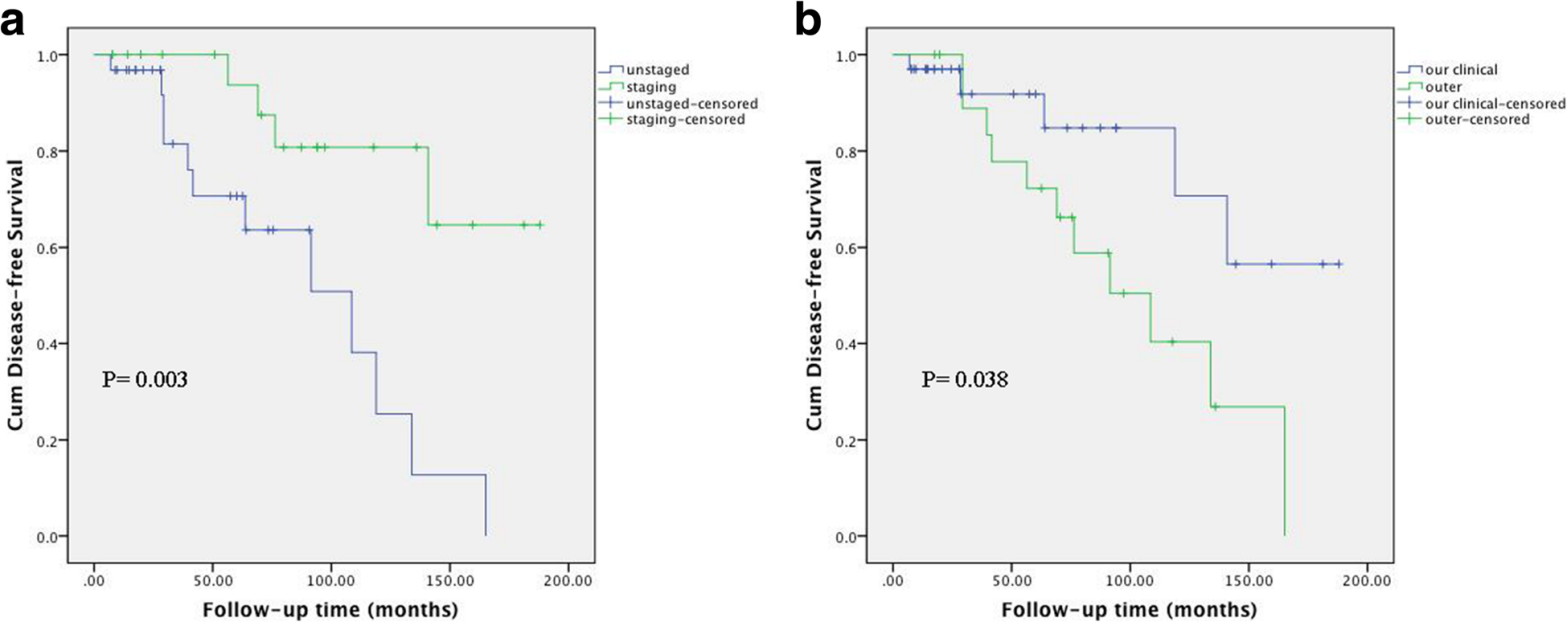
Disease-free survival according to staging surgery (**a**) and initial treatment place (**b**)

**Fig. 2:**
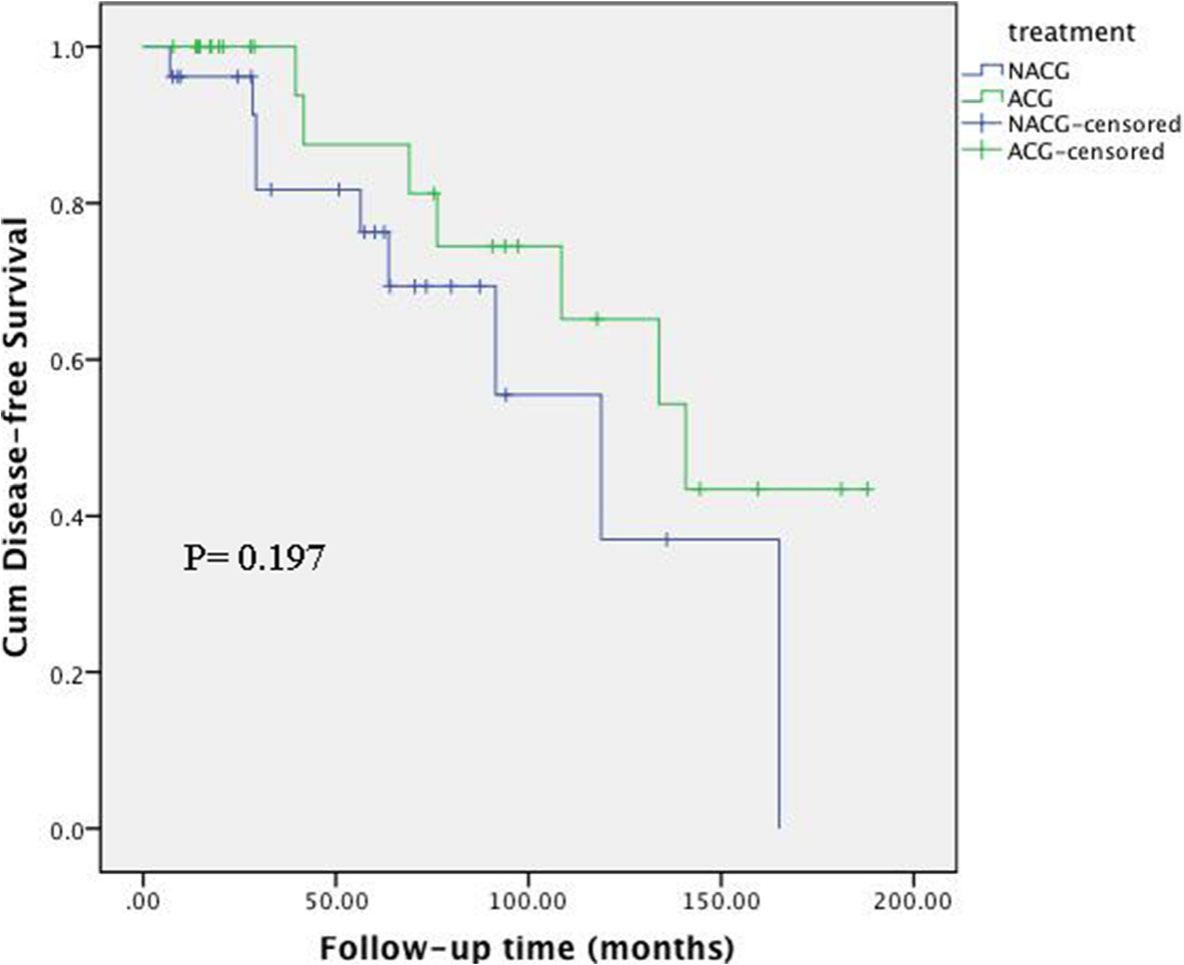
Disease-free survival according to receiving adjuvant chemotherapy or not. NACG: no adjuvant chemotherapy group; ACG: adjuvant chemotherapy group

**Fig. 3:**
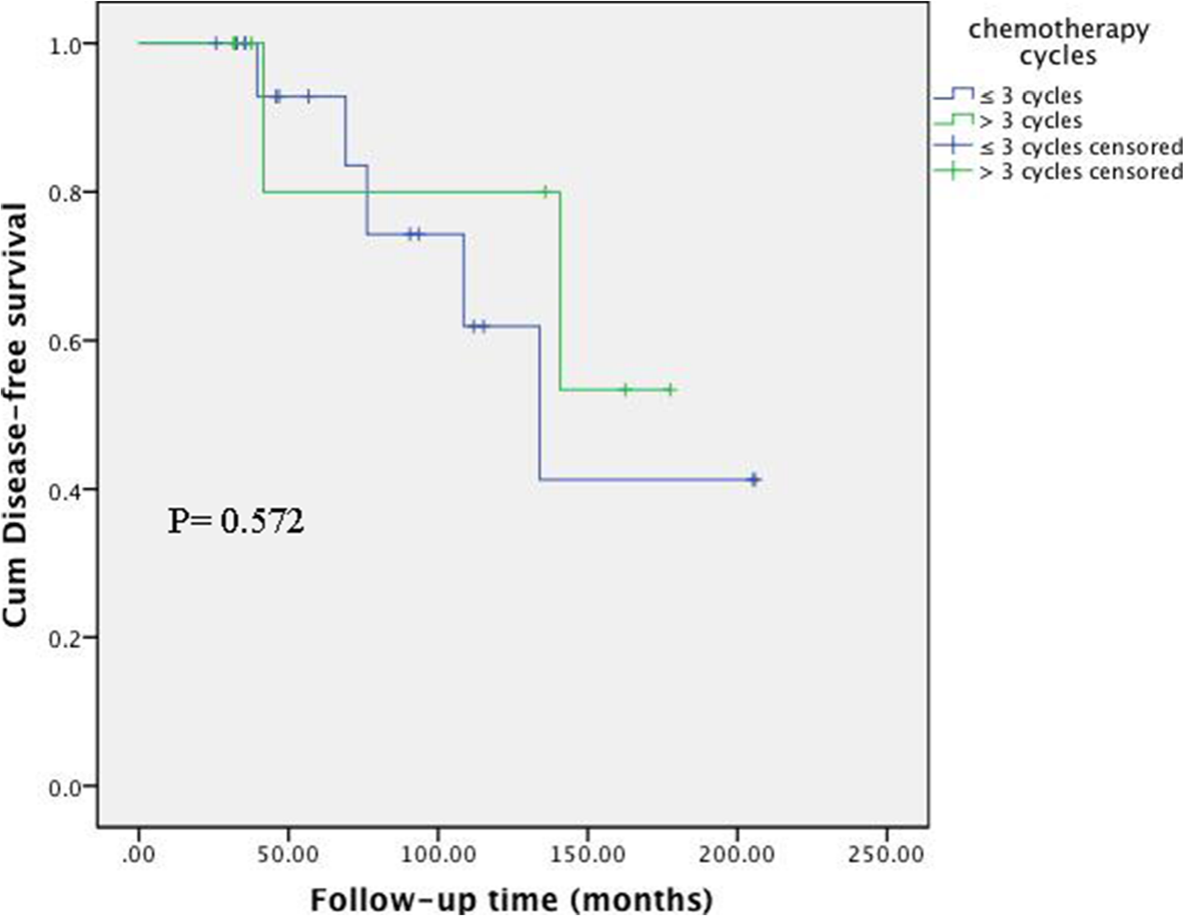
Disease-free survival according to the number of chemotherapy cycles in 32 patients who received adjuvant chemotherapy

The associations between clinical factors and DFS in the 60 patients with stage IC AGCT are shown in Table 2. According to univariate analysis, menopause, FIGO stage, adjuvant chemotherapy, and lymph node dissection were not associated with DFS, while surgical staging (*P* = 0.003) and initial treatment place (*P* = 0.038) were significantly associated with DFS (Fig. 1). Incomplete surgical staging (hazard ratio (HR) = 3.883, 95% confidence interval (CI): 1.123–13.430, *P* = 0.032) was still a significant predictive factor for recurrence in multivariate analysis. The 5-year DFS rates in the NACG and ACG groups were 76.3% and 87.5%, respectively (*P* = 0.197) (Fig. 2). Further analysis of the ACG subgroups revealed no association between the number of chemotherapy cycles and the incidence of recurrence (≤3 cycles vs. > 3 cycles, HR = 0.613, 95% CI: 0.112–3.351, *P* = 0.572) (Fig. 3).

**Table 2:** Univariate and multivariate analysis of patients in stage IC AGCT

Factors	5 year DFS rate (%)	Univariate		Multivariate	
		HR (95% CI)	P	HR (95% CI)	P
Menopause			0.065		
Yes	100	1			
No	75.4	0.183 (0.024–1.391)			
Surgery			0.003		0.032
Staging	93.8	1		1	
Unstaged	70.6	4.95 (1.557–15.792)		3.883 (1.123–13.430)	
Stage			0.971		
IC1	76.9	1			
IC2-IC3	80.8	1.019 (0.368–2.819)			
Adjuvant chemotherapy			0.197		
No	76.3	1			
Yes	87.5	0.517 (0.186–1.433)			
Initial treatment			0.038	0.345	0.345
Our clinical	91.9	1		1	
Outer	72.2	2.984 (1.012–8.799)		1.747 (0.549–5.562)	
Lymph node dissection			0.386		
Yes	87.8	1			
No	76.1	1.578 (0.557–4.472)			

AGCT: adult granulosa cell tumor; DFS: disease-free survival

## Discussion

Adjuvant chemotherapy was not associated with improved DFS in the current cohort of 60 patients with stage IC AGCT. Furthermore, the number of cycles of chemotherapy was not associated with improved DFS among those patients who received postoperative chemotherapy.

AGCT is a late-relapse disease, and long-term follow-up is necessary to obtain reliable data [12]. The median follow-up time in our study was significantly longer than in other recent reports (88 months; range: 9–334 months) [3, 5], and the recurrence rate was 26.7%, which was consistent with previous reports [4, 13], suggesting that this was a realistic and representative reflection of the natural history of the disease.

NCCN guidelines suggest that adjuvant chemotherapy should be considered in patients with early-stage disease but with high risk factors (e.g., high mitotic index, tumor rupture, or incomplete surgical staging); however, our data do not appear to support this recommendation, and showed that adjuvant chemotherapy did not protect against recurrence in patients with AGCT. Adjuvant chemotherapy is not always administered in our practice, and almost half (46.7%) of all patients with stage IC disease did not receive adjuvant chemotherapy. However, the retrospective nature of the trial means that the reasons why these patients did not receive adjuvant chemotherapy were unknown. Previous studies have provided conflicting reports regarding the role of adjuvant chemotherapy in AGCT. Several studies showed beneficial effects of platinum-based treatments [3, 14, 15], though most of these included patients with advanced stage disease, which has a poorer prognosis than early stage disease, and unadjusted survival analyses meant that the role of chemotherapy in stage I disease remained obscure. However, adjuvant chemotherapy was not associated with a reduced recurrence rate, even when the survival analysis was restricted to patients with stage I AGCT [6, 12]. Mangili et al. also recently reported no difference in DFS between stage IC patients with or without adjuvant chemotherapy [11]. The potential toxicity of chemotherapy, including second acute leukemia [16] and cardiovascular disease [17], together with a lack of obvious benefit, suggest that more studies are needed to clarify the benefit and thus inform the decision to administer chemotherapy for early stage AGCT.

BEP is the most widely used first-line adjuvant therapy regimen among patients for whom adjuvant therapy has been deemed appropriate [18]. However, these agents are associated with potentially serious toxicities, such as myelosuppression and pulmonary disorders associated with bleomycin [19]. Prospective trials are therefore needed to assess the therapeutic ratio of regimens other than the widely used BEP. NCCN guidelines (category 2B) also recommend TC, and the Gynecologic Oncology Group is currently developing a randomized phase II trial to compare TC with BEP, with progression-free survival as the primary outcome (ClinicalTrials.gov Identifier NCT01042522). The expectation is that TC may be associated with reduced toxicity and similar progression-free survival compared with BEP. BEP and TC regimens were both used in some of our patients; however, the retrospective nature of the study and the variety of chemotherapy regimens and doses used means that it was difficult to draw any conclusions regarding the relative values of the different regimens in this study.

Age, FIGO stage, staging surgery, and initial treatment place have been reported as prognostic factors in AGCT [3, 12]. Surgical staging was associated with improved outcomes in the present series, with 5-year DFS rates of 93.8% and 70.6% in patients with and without complete staging, respectively (*P* = 0.003). These results were consistent with previous studies demonstrating that the recurrence rate was significantly increased in incompletely staged patients. In Park’s report, completely staged patients had no recurrence or deaths, while recurrence was observed in 14.3% of patients without complete staging [3]. Seagle et al. analyzed prognostic information from a national cancer database and found that incomplete surgical staging was associated with increased risk of death [20]. These reports highlight the need for an accurate diagnosis at presentation.

Initial treatment outside was a risk factor for recurrence in univariate analysis (5-year DFS rate: 91.9% vs 72.2%), though this was not a significant factor in multivariate analysis. This may be explained by suboptimal surgical extent, delayed initiation of adjuvant treatment in high-risk patients, or an inaccurate pathological diagnosis.

This study raises questions over the importance of chemotherapy in patients with stage IC AGCT. The lack of any obvious benefit of adjuvant chemotherapy suggests that new effective treatment modalities are needed, based on a deeper understanding of the pathogenesis of AGCT. Shah et al. examined *FOXL2* gene mutations in ovarian granulosa cell tumors [21] and showed that *FOXL2* mutation was associated with increased CYP17 expression. Inhibition of this enzyme may thus reverse the effect of *FOXL2* mutation. Additional inhibition of CYP17 may be achieved by novel agents such as abiraterone and ketoconazole [22], and therapies targeting this mechanism may prove to be more useful and less toxic than traditional chemotherapy [5]. Other novel potential targets (vascular endothelial growth factor (VEGF), HER2) are being investigated. In an era of precision medicine, postoperative patient care and decisions about adjuvant chemotherapy can be individualized based on these immunohistochemical factors, such that patients whose GCTs show high expression levels of VEGF or HER2 can be treated with bevacizumab or trastuzumab/imatinib, respectively [23, 24].

The current study was limited by its retrospective nature, including heterogeneity in terms of staging and work-up, and variable chemotherapy regimens, as well as by the rarity of the disease. However, the present study included a relative large cohort of stage IC patients and the results thus contribute to the limited body of knowledge on this condition. Further studies in large series are needed to characterize patients with stage IC subtype who can be spared adjuvant chemotherapy, and to define the real risk factors in stage IC. However, prospective clinical trials are difficult and time-consuming given the rarity of the disease, its indolent nature, and its overall good prognosis in the early stage. International collaboration is therefore needed to generate large studies with the aim of validating new treatment regimens for patients with high-risk early stage AGCT.

## Conclusions

Adjuvant chemotherapy does not improve DFS in patients with stage IC AGCT. Further clinical trials, including using novel agents, are needed to characterize patients with stage IC AGCT who can be spared adjuvant chemotherapy, and to define the real risk factors in this disease.

## Abbreviations

ACG: Adjuvant chemotherapy group
AGCT: Adult granulosa cell tumor
BEP: Bleomycin, etoposide and cisplatin
DFS: Disease-free survival
FIGO: International Federation of Gynecology and Obstetrics
FOXL2: Forkhead Box L2.
HER2: Human epidermal growth factor receptor-2.
NACG: No adjuvant chemotherapy group.
NCCN: National ComprehensiveCancer Network.
PVB: Cisplatin, vincristine, bleomycin.
PC: Cisplatin, cyclophosphamide.
TC: Paclitaxel and carboplatin.
VEGF: Vascular endothelial growth factor.
